# Acute hepatitis of unknown origin in Europe—Adding fuel to already burning pandemic

**DOI:** 10.1016/j.amsu.2022.104392

**Published:** 2022-08-18

**Authors:** Maliha Tahir, Sejal Lund, Amatul Hadi Hamdana, Shahzaib Ahmad, Muhammad Umar, Shahzaib Farid, Muhammad Osama Siddiqui, Muhammad Muneeb Khawar

**Affiliations:** a Mayo Hospital, Lahore, Pakistan; b Shaheed Mohtarma Benazir Bhutto Medical College, Karachi, Pakistan; c Dow Medical College, Karachi, Pakistan; d Khairpur Medical College, Khairpur, Pakistan; e Liaquat National Hospital and Medical College, Karachi, Pakistan

**Keywords:** Acute hepatitis, Children, Liver transplantation, Virus, Unknown origin

## Abstract

The rise in the cases of acute hepatitis of unknown aetiology in the paediatric population is a public health concern worldwide and investigations to ascertain the exact cause of this outbreak are being carried out extensively by the concerned authorities. In early April 2022, the World Health Organisation (WHO) issued a warning on acute hepatitis of unknown origin in children. Since then, there have been continuing additional reports of the cases globally. The recent cases of acute hepatitis of unknown aetiology are more prevalent in children aged <10 years, are more clinically severe, and a high percentage of infected individuals develop acute liver failure in contrast to the previous cases. The aetiology of this disease and its complete pathogenesis is still unclear. This review critically focuses on the current leading hypothesis and provides comprehensive information regarding this recent outbreak that can help in handling the situation by a better understanding of its aetiology.

## Highlights

• Various cases of acute hepatitis of unknown origin have been reported to WHO from different countries.
• Most of the cases reported were of the children from one month to 16 years and majority of them were infected with adenovirus.
• The diseases is challenging due to its unknown aetiology, insufficient data to establish management plans and its severity.
• International health organizations have teamed up and set up surveillance systems and investigations to help identify the aetiology.
• WHO and CDC have recommended general preventive measures including hand and respiratory hygiene.

## 1 Introduction

Recently, the COVID-19 pandemic has caused chaos across the world. In the face of an ongoing pandemic, an epidemic of acute hepatitis of unknown origin is feared to further exacerbate the situation. The World Health Organization (WHO) and The European Center for Disease Prevention and Control (ECDC) have presented a common case definition defining a probable case of acute hepatitis of unknown origin as a person presenting with acute hepatitis (non-hepatitis viruses A, B, C, D, and E) with serum transaminase levels >500 IU/L (AST or ALT), who is 16 years and younger, since October 1, 2021 [1,2]. 650 probable cases of hepatitis of unknown origin in children were reported to WHO between 5th April and May 26, 2022, from 33 countries in five WHO Regions. Children between the age of 1 month–16 years are typically infected. At least 38 children required liver transplantation and nine deaths have been reported to WHO till now [1]. As of June 9, 2022, 402 cases of acute hepatitis of unknown origin in children aged 16 years and below have been reported from the European region. The majority of these cases were five years old or younger [2]. The aetiology of this severe acute hepatitis is unknown; the cases are clinically more severe, and a higher proportion develops acute liver failure, as compared to earlier reports of acute hepatitis in children with an unknown aetiology [1]. Adenoviruses have become the focus of investigations after a large number of cases were found to be infected with them [3]. The majority of cases were observed to present with gastrointestinal symptoms such as stomach discomfort, diarrhea, and vomiting, followed by the appearance of jaundice [4]. As a result of the COVID-19 pandemic, the world is going through an economic crisis. According to 2020 estimates, the Gross Domestic Product (GDP) of Europe has declined by 6.1%, more than the global financial crisis. Now this outbreak of acute hepatitis in multiple countries in Europe may aggravate the already existing economic crisis, which in turn may result in a negative impact on health systems and health budgets in those countries [5]. The aim of this review is to shed light on the current status of this outbreak of acute hepatitis of unknown origin in children, its probable aetiology, and the roles played by healthcare workers and different organizations in understanding its aetiology, epidemiology, and the possible measures that can assist in halting the spread of this outbreak.

## 2 Signs and symptoms

The affected individuals most commonly present with jaundice (yellowing of the skin and eyes) (68.8%), followed by vomiting (57.6%). Patients also present with lethargy (48.6%), malaise, fatigue, loss of appetite, diarrhea (43.1%), dark urine, light-colored stools (42.7%), fever (28.5%), abdominal pain (26.1%), nausea (25.7%), and respiratory symptoms (18.1%). According to the recent studies, eleven of the patients in the UK suffering from acute hepatitis of unknown origin showed severe manifestations including end-stage liver failure requiring liver transplant [[6], [7], [8]].

### 2.1 Aetiology

According to the UK Health Security Agency (UKHSA), tests for hepatitis viruses A-E and other known causes of acute hepatitis have been universally negative in the recent reported cases of patients infected with acute hepatitis of unknown origin. However, intricate laboratory testing has found that 72% of the children infected with acute hepatitis tested positive for human adenovirus (HAdV). Adenoviruses are DNA viruses that usually cause mild infections of the respiratory tract, gastrointestinal tract, or conjunctiva. The cases are more common in young children due to lack of immunity and are typically self-limiting. Adenovirus type 41 was identified in blood samples of some children [[9], [10], [11]]. The Alabama Department of Public Health (ADPH) has been studying a rise in hepatitis in young children throughout the state of Alabama since November 2021, in partnership with paediatric healthcare providers, including hospitals that treat children, and the Centers for Disease Control and Prevention (CDC). Their investigations identified a possible link between this hepatitis and Adenovirus 41 [12]. The UKHSA, in its 3rd technical briefing on investigating the details of acute hepatitis of unknown aetiology in children, has outlined various hypotheses that are being actively tested for the origins of this outbreak. These hypotheses include [8]:

● A normal adenovirus with an exceptionally large wave of normal adenovirus infections causing a very rare or under-recognised complication to present more frequently.
● An abnormal susceptibility or host response to adenovirus due to priming by a prior infection with SARS-CoV-2 or another infection.
● An abnormal susceptibility or host response to adenovirus due to a coinfection with SARS-CoV-2 or another infection.
● An abnormal susceptibility or host response to adenovirus due to a toxin, drug, or environmental exposure.
● A novel variant of adenovirus, with or without a contribution from a cofactor as listed above.
● A post-infectious SARS-CoV-2 syndrome, some drug, toxin or environmental exposure, a novel pathogen either acting alone or as a coinfection, or a new variant of SARS-CoV-2.

Recent studies have shown Adenovirus to be the most probable cause of acute hepatitis, as 55% and 75% of acute hepatitis cases in England and Scotland, respectively, and 68% of cases in the UK, have tested positive for adenovirus [7,8]. Current investigations to discover the real cause of this outbreak of acute hepatitis of unknown origin are still ongoing.

## 3 Challenges

The upsurge in the cases of acute hepatitis of unknown origin in the paediatric population poses a threat to global public health. As of June 9, 2022, 402 cases of acute hepatitis of unknown cause have been reported in Europe [13]. The major challenges related to the disease are mainly due to its unknown aetiology. Detailed questionnaires were employed in the United Kingdom early in the outbreak to evaluate the eating, drinking, and personal habits of the patients, but they failed to identify a common exposure between cases. In the absence of data suggesting a toxicologic or environmental cause, health officials believe the current acute hepatitis outbreak is most likely caused by an infectious agent, but toxicologic factors are yet to be ruled out [14]. Although 75% of the cases in the UK and more than half of the cases in the US have tested positive for adenovirus, the data is still incomplete [1,15]. According to WHO, the recent cases being reported are more clinically severe and are associated with acute liver failure (ALF). Even though most countries have the medical resources to treat acute hepatitis, severe cases that lead to indeterminate ALF require intensive care support and liver transplantation, and these facilities are not easily available in every country [1]. Knowledge regarding the origin and pathogenesis of the disease is vital to establishing a treatment plan and managing other associated and underlying disorders. WHO and ECDC have also not yet been able to develop specific guidelines for the prevention and control of disease since the source and route of transmission and predisposing factors are not known with certainty [1,14].

## 4 Current efforts

Recently, due to the increase in the cases of hepatitis of unknown origin among the children across Europe, the United States Of America, Israel, and Japan, the Centers for Disease Control and Prevention (CDC) and the European Center for Diseases Prevention and Control (ECDC) have issued a warning of a sudden outbreak [1,7]. ECDC and the World Health Organization (WHO), in collaboration with all those countries that have reported these acute hepatitis cases, are investigating the aetiology of this outbreak [1]. ECDC and WHO have also set up a surveillance system based on the case definition, which is, “A person presenting with acute hepatitis (non-HepA-E) with serum aminotransferases >500 IU/L (AST or ALT), who is 16 years and younger, since 1st October 2021.” [7] According to the surveillance system, cases similar to the case definition should be reported to the European Surveillance System (TESSy) [9]. Furthermore, a juncture of WHO/ECDC has been set up using the European Surveillance System (TESSy) [7]. Various investigations including toxicology testing i.e., environmental and food toxicity testing, virological and microbiological testings are being carried out by the affected countries to help identify the causative agent [1]. Public health officials have raised awareness of the disease via social media and other outlets by alerting the general public, especially parents, to look out for any signs and symptoms of hepatitis (jaundice, yellow tinge in the whites of the eyes, abdominal pain, vomiting, pale stools) in their children. Moreover, health officials also often respond to any misinformation spreading on social media to prevent the occurrence of an infodemic during the outbreak [14,16].

The European Association for the Study of Liver (EASL) is attempting to raise awareness about the cases and is working closely with the European Centre for Disease Prevention and Control (ECDC) to investigate the causes and characteristics of the cases. Their major concern is for the critical cases of acute hepatitis that underwent liver transplants and hospital admissions. Acute hepatitis in children is more complicated than just one infectious agent. According to ECDC, there are few cofactors that are responsible for the most severe kind of acute hepatitis of unknown origin: infection with SARS-CoV-2, exposure to a toxin, or environmental exposure [17].

According to the UKHSA third briefing on the investigations into acute hepatitis of unknown aetiology in children in England, UKHSA is working on the investigations on analytic epidemiology and surveillance of the problem, investigations on the mechanism of liver injury are underway by the NHS, associated pathogen investigations are being carried out by the UKHSA, Great Ormond Street Hospital (GOSH), International Severe Acute Respiratory and emerging Infection Consortium (Comprehensive Clinical Characterisation Collaboration) (ISARIC4C), and academic partners, while ISARIC4C in partnership with Genetics of susceptibility and Mortality in Critical Care (GenOMICC) is working on investigating the host characterisation [8].

## 5 Recommendations

As adenovirus is suspected to play a role in the aetiology of acute hepatitis of unknown origin, measures to protect against the spread of adenoviruses should be emphasized. Adenovirus can spread through close personal contact with an infected individual, and it is also resistant to common disinfectants [16,18]. Public healthcare authorities advise frequent handwashing, avoiding touching the nose, eyes, and mouth with unwashed hands, avoiding close contact with anyone who is sick, and sterilizing surfaces as the fecal oral route is the most likely mode of transmission in children, especially those infected with HAdV 41 [1,14,19]. Since the aetiology of these acute hepatitis cases is not confirmed yet, WHO has established general infection prevention and control guidelines including avoiding crowded spaces and maintaining a distance from others, ensuring good ventilation when indoor, wearing a well-fitted mask covering your mouth and nose when recommended, covering coughs and sneezes, using safe water for drinking, following safe food handling and cooking practices, staying home when unwell and seeking medical attention [1]. We recommend that digital and social media platforms be used to make the general public aware of these precautionary measures. ECDC recommends that paediatricians, general practitioners, and other medical specialists should be informed by public health authorities about the importance of aggressive case finding and reporting of new cases. Adenoviruses, as well as other viruses that can cause hepatitis, should be regularly tested in relevant samples from sick children as soon as possible after symptoms appear. To identify the causative agent or co-factors, the ECDC advises a comprehensive series of tests [9]. Screening through polymerase chain reaction (PCR) is advised for adenovirus detection and testing whole blood by PCR is preferred against testing for plasma by PCR [20]. Cases that meet the case definition should be reported as soon as possible to TESSy [9]. The worldwide response to mitigate the situation can be aided by a rigorous and empirical strategy that uses standardized case definitions as already developed by WHO and ECDC, diagnostic methods, efficient communication, and collaboration [1,2,10]. Future research is needed to determine the exact cause of this hepatitis of unknown origin in children to aid in the treatment and prevention of this disease.

## 6 Conclusions

In conclusion, amidst the COVID-19 pandemic, the multicountry outbreak of hepatitis of unknown origin is a new challenge to the healthcare community around the globe, especially due to its unknown aetiology. So far, human adenovirus infection has been identified as the most likely cause, but it still remains unconfirmed. Due to the limited understanding of the pathogenesis of acute hepatitis of unknown origin, no specific treatment or vaccines are available yet. The most urgent task at the moment is to identify the origin and mode of transmission of the disease. Furthermore, the development of more interventions is necessary to effectively control the rise in the number of cases. Until then, WHO and ECDC general infection control guidelines should be followed.

## Ethical approval

Not required.

## Sources of funding

No source of funding

## Author contribution

All authors contributed substantially to all the steps involved in writing this article including study concept and design, writing the paper, and critical review.

## Registration of research studies

Not applicable.

## Guarantor

Dr. Maliha Tahir, Email address: malihatahir15@gmail.com.

## Declaration of competing interest

No conflicts of interest.
